# Domino-like transient dynamics at seizure onset in epilepsy

**DOI:** 10.1371/journal.pcbi.1008206

**Published:** 2020-09-28

**Authors:** Jennifer Creaser, Congping Lin, Thomas Ridler, Jonathan T. Brown, Wendyl D’Souza, Udaya Seneviratne, Mark Cook, John R. Terry, Krasimira Tsaneva-Atanasova

**Affiliations:** 1 Department of Mathematics, University of Exeter, Exeter, EX4 4QF, UK; 2 EPSRC Centre for Predictive modeling in Healthcare, University of Exeter, Exeter, EX4 4QJ, UK; 3 Center for Mathematical Sciences, Huazhong University of Science and Technology, Wuhan, Hubei 430074, China; 4 Hubei Key Lab of Engineering Modeling and Scientific Computing, Huazhong University of Science and Technology, Wuhan, Hubei 430074, China; 5 Living System Institute, University of Exeter, Exeter, EX4 4QJ, UK; 6 Institute of Biomedical and Clinical Sciences, College of Medicine and Health, University of Exeter, EX4 4PS, UK; 7 Department of Medicine, St. Vincent’s Hospital, University of Melbourne, Melbourne, VIC 3065, Australia; 8 Department of Neuroscience, Monash Medical Centre, Melbourne, VIC 3168, Australia; 9 Graeme Clark Institute, University of Melbourne, Parkville, VIC 3010, Australia; 10 Centre for Systems Modelling and Quantitative Biomedicine, University of Birmingham, Birmingham, B15 2TT, UK; 11 Institute for Advanced Study, Technical University of Munich, Lichtenbergstrasse 2a, D-85748 Garching, Germany; 12 Department of Bioinformatics and Mathematical Modelling, Institute of Biophysics and Biomedical Engineering, Bulgarian Academy of Sciences, 105 Acad. G. Bonchev Str, 1113 Sofia, Bulgaria; Institut de Neurosciences des Systèmes, FRANCE

## Abstract

The International League Against Epilepsy (ILAE) groups seizures into “focal”, “generalized” and “unknown” based on whether the seizure onset is confined to a brain region in one hemisphere, arises in several brain region simultaneously, or is not known, respectively. This separation fails to account for the rich diversity of clinically and experimentally observed spatiotemporal patterns of seizure onset and even less so for the properties of the brain networks generating them. We consider three different patterns of domino-like seizure onset in Idiopathic Generalized Epilepsy (IGE) and present a novel approach to classification of seizures. To understand how these patterns are generated on networks requires understanding of the relationship between intrinsic node dynamics and coupling between nodes in the presence of noise, which currently is unknown. We investigate this interplay here in the framework of domino-like recruitment across a network. In particular, we use a phenomenological model of seizure onset with heterogeneous coupling and node properties, and show that in combination they generate a range of domino-like onset patterns observed in the IGE seizures. We further explore the individual contribution of heterogeneous node dynamics and coupling by interpreting in-vitro experimental data in which the speed of onset can be chemically modulated. This work contributes to a better understanding of possible drivers for the spatiotemporal patterns observed at seizure onset and may ultimately contribute to a more personalized approach to classification of seizure types in clinical practice.

## Author summary

Epilepsy is a serious neurological condition encompassing a variety of syndromes that affect around 65 million people worldwide. Seizure type in epilepsy is characterized by onset pattern and brain network involved into three categories that do not fully capture the complexity of observed onset patterns. Ambiguity of seizure onset observed in the clinic could result in significant diagnostic delay and inappropriate treatment for an individual. We show how a variety of recruitment patterns across a network arise as the result of interplay between heterogeneous node dynamics and heterogeneous coupling among nodes. Our results demonstrate the important role of brain network dynamics in driving spatiotemporal patterns of seizure onset and provide a dynamic mechanism that could inform novel classifications of seizure types in clinical practice.

## Introduction

Recurrent spontaneous seizures, the hallmark of epilepsy, are characterized by behavioral symptoms alongside abnormal patterns of electrical activity in the brain. These are believed to be caused by an imbalance between excitation and inhibition within neural populations leading to hyper-excitability at the macro scale [1].

The International League Against Epilepsy classification of epilepsy 2017 classifies seizures by type of onset and brain networks affected. A seizure initiated in a local brain region is classified as focal, while a seizure involving activation of brain regions on both sides of the brain is classified as generalized. In the case when the above is not clear or known, a seizure is classified as unknown [2, 3]. This division represents a practical separation made on clinical grounds and (where possible) informed by electroencephalogram (EEG) recordings in order to guide treatment. By lumping together a variety of seizure onset patterns into these three broad categories, the diversity of spatiotemporal patterns observed at seizure onset, for example, focal onset of generalized seizures, are typically overlooked. Note that focal onset of a generalized seizure is simply an EEG variation seen in idiopathic generalized epilepsy (IGE) but not an indication of focal epilepsy [4]. However, ambiguity of seizure onset captured in the clinic could result in significant diagnostic delay as well as unnecessary invasive investigations and inappropriate anti-epileptic drug therapy in some cases.

Despite recent advances, there remains insufficient knowledge for a purely scientific classification of seizure types [2, 3, 5]. Current research identifies seizures as the initiation and recruitment of neuronal populations in distinct but interconnected brain regions. Brain activity can thus be modeled by means of a dynamic network in which each node represents a population of neurons in a specific region of the brain. Understanding the spatiotemporal dynamics driving recruitment of nodes from background to seizure state across a given network is crucial to inform and advance scientific taxonomy of seizure onsets. Local and global changes in functional and structural network properties generate “hyper-excitable” networks, characterized by high frequency switches from background to seizure states [6]. However, the relative contribution to the network level activity from individual or populations of nodes is not well understood.

In this paper, we identify and investigate a variety of domino-like onset patterns observed in EEG recordings in IGE and characterize them by total recruitment time (i.e. the time it takes for seizure activity detected in one electrode to spread to all electrodes) and the lag time between recruitment of sequential electrodes. We show that different patterns of detected activity are clinically observed in the same individual. We develop a framework for modeling seizure onset and use it to demonstrate how different onset patterns arise as a result of interplay between heterogeneous node dynamics and heterogeneous coupling between nodes. We then explore the individual contribution of heterogeneity in node excitability and coupling among nodes.

To this end, we use a phenomenological network model of seizure onset previously studied by us and others [7–11]. Each node in the network has two stable states: background (steady) and seizure (oscillatory). The transition between the two is driven by an external random ‘noisy’ input. The same model has been used to explore domino-like recruitment of nodes across motif networks [7, 9]. Each node is set to the background state (domino standing upright on a table) and transits to the oscillatory state (domino lying down on a table) due to noisy perturbations and inputs from other nodes (the table is wobbled). Depending on how likely it is to fall over (intrinsic property) or how close to other dominoes it is (coupling) you get slow or fast toppling patterns. We show that the domino-like seizure onset patterns observed in the EEG data have fast-domino and slow-domino cascades. Moreover we extend this approach by introducing the novel multiple-domino onset where large lags (gaps) separate groups of sequential electrodes for which the detected activity has similar transition times.

We build on previous work by considering larger networks and introduce heterogeneity to the node excitability and coupling weights. We first apply the model to generalized seizures observed in EEG recordings that exhibit fast-, slow- and multi-domino onset patterns. To illustrate our findings, we present in detail the analysis of a single individual with Juvenile Absence Epilepsy, taken from the data set detailed in [4, 12]. Secondly, to unambiguously separate the contribution of node excitability or coupling weight to the observed onset patterns requires a tractable and controllable in-vitro model. To this end, we also apply our modeling framework to the slow onset of seizure-like activity induced along slices of medial entorhinal cortex (mEC) from mice, detailed in [13]. Our results highlight the complex interplay between network properties and provide a compelling case for further investigation into their impact on emergent dynamics in order to better categorize seizures and advance patient-specific diagnosis and treatment.

## Materials and methods

### Clinical EEG data

We use epileptiform events recorded using EEG from patients diagnosed with genetic generalized epilepsy (GGE). Full details of the database can be found in [4, 12]; for completeness we summarize briefly. Ambulatory EEG (Compumedics Ltd, Melbourne, Australia) were recorded from 107 patients for a period of 24-hours using 32 gold cup electrodes arranged according to the international 10–20 system. Signals were recorded with a sampling rate of 256Hz. The ProFusion 4 software (Compumedics Ltd, Melbourne, Australia) was used by an experienced EEG reader to review the recordings, identify and extract epileptiform events. Patients were categorized into five groups depending on syndrome: childhood absence epilepsy (CAE); juvenile absence epilepsy (JAE); juvenile myoclonic epilepsy (LME); generalized epilepsy with generalized tonic-clonic seizures only (GTCSO); and genetic generalized epilepsy unspecified (GGEU). Data was manually annotated by the clinician based on the common average reference [4, 12, 14]. The data set consists of 15 second long epochs each containing an event positioned such that the clinician identified onset time is at 5 seconds. Events from recordings were classified into three types: generalized paroxysms (events lasting longer than 2s), generalized fragments and focal discharges. The onset of generalized paroxysms was further classified into generalized onset or focal onset by a trained EEG reader. Focal onset of a generalized paroxysm is defined as lead-in focal discharges lasting at least 0.2s [12].

In this paper, we consider epochs containing generalized paroxysms, that we will henceforth call seizures. We extract 1267 epochs from the database containing seizures from 59 patients, including 15 seizures classified as focal onset. Note that there are no epochs containing seizures in the GGEU group. We submit each 15s epoch to the seizure onset detection algorithm, detailed below. We illustrate the application of the modeling framework using three consecutive seizures from one person with JAE, which is the most common syndrome within the data set [12].

### Mouse mEC recording

**Fig 1:**
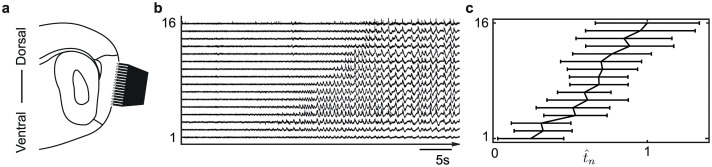
Multi-electrode recording from mouse mEC. Panel **a** shows an illustration of the silicone probe positioned along the dorsal-ventral axis of the mEC. Panel **b** is an example seizure-like onset from mEC data showing the slow domino effect, where sequential seizure-line onset starts in the ventral end and spreads to the dorsal. Panel **c** is the scaled mean recruitment times ${\hat{t}}_{n}$ and standard error for each electrode *n* = 1, …, 16 from six seizure-like events.

Detailed description of the procedures used to collect recordings and analyze the data from the mouse mEC are given in [13]. All procedures were carried out in accordance with the UK Animal (Scientific Procedures) Act 1986 and were approved by the University of Exeter Animal Welfare and Ethical Review Body. Here we provide a brief summary for completeness. Parasagittal brain slices (400 *μ*m thick) containing mEC were collected from male C57/BL6 mice. The slices were transferred to an interface-style recording chamber, and allowed to equilibrate for 30 minutes before a bath application of 4-aminopyridine (4-AP; 100 *μ*M concentration equilibrated). A silicone probe consisting of 16 individual shanks (55 *μ*m wide, 100 *μ*m apart), with a single electrode contact point at the end of each shank (Neuronexus, Ann Arbor, MI; probe catalog number: A16x1-2mm-100-177), was positioned along the dorsal-ventral axis of the mEC; see Fig 1. A 32-channel amplifier (RHD2132; Intan, Los Angeles, CA) coupled to an open-source acquisition board (Open Ephys Inc, Cambridge, MA) was used to take recordings and the data were subsequently band-pass filtered (1-500 Hz) and digitized at 2 kHz.

In this paper, we apply our modeling framework and analysis to six recordings of length between 18 to 50 min, each containing several ‘seizure-like’ events. From each recording we determine the first onset event in which all 16 channels transit to a seizure-like state; this occurs 9-20 min from the start of the recordings using the same threshold detection algorithm (detailed below) to determine onset in each channel.

In further experiments, the induction of epileptiform activity by bath application of 4-AP was followed by co-application of GABA_A_ receptor modulators as either positive modulation via diazepam (30 *μ*M) or negative with Ro19-4603 (10 nM) [15]. In a final set of experiments considered here, a scalpel blade was used to make a cut in the intermediate mEC, thus anatomically separating dorsal and ventral portions. In this case, data were recorded from two sites, one in the dorsal and one in the ventral end of the slice; the 16 channel multielectrode array was not used in this case [13].

### Seizure onset detection

We refer to the moment of transition from background to seizure state as *seizure onset time*. It is important to note that we detect this change in activity in the same way in both the EEG and mEC data sets, using a threshold detection algorithm as in [13] implemented using custom code in Matlab (ver. R2018b).

The threshold detection algorithm is as follows:

1. The signal from a single channel or electrode is band pass filtered then z-normalized followed by rectifying, giving the processed signal *x*(*t*) for each time point *t*.
2. An envelope *u* is determined using spline interpolation over local maxima separated by at least *np* data points.
3. *Seizure onset time* *τ*_*n*_ for each electrode *n* is the first time over a threshold defined by *s*×standard deviation from the mean of the envelope,
  $$\begin{array}{c} \tau_{n}≔\text{min}\left\{t:|x\left(t\right)|>\text{mean}\left(u\right)+s*\text{std}\left(u\right)\right\}. \end{array}$$

The choice of band-pass filter, *np* and *s* values is different in the two cases (human and animal) and depends on the sampling frequency and seizure duration time in each data case. The values used in this paper are given in Table 1 and the algorithm procedure is illustrated in S1 Fig.

**Table 1:** The values used in the threshold detection algorithm for the two types of data used to obtain the results presented in this paper.

	EEG data	mEC data
band pass filter	4-20Hz [16]	15-35Hz [13]
*np*	60	22000 [13]
*s*	0.6	1 [13]

For the EEG data, we submit 1267 epochs (described above) to the threshold detection algorithm. We only consider epochs in which onset is detected in all electrodes for further analysis; onset is detected in all electrodes in 1159 seizures out of the total 1267. From the detected onset times for each seizure we calculate the *recruitment time* defined as *t*_*n*_ = *τ*_*n*_ − min(*τ*_*n*_) for each electrode *n*. We order the electrodes for each seizure by recruitment time from min(*t*_*n*_) = 0 to max(*t*_*n*_). The *total recruitment time* is max(*t*_*n*_) and we calculate the *maximum lag* by taking the largest difference between consecutive (ordered) recruitment times. Note here that we are interested in the diverse range of patterns observed at seizure onset and that the seizures are recorded using ambulatory EEG so may occur under different (unknown) conditions e.g. sleep/wake. Therefore, we do not average recruitment times over seizures and instead submit them to a linear discriminant analysis detailed in the next section.

In the case of mouse data we submit the six manually identified mEC epochs containing seizure-like events to the threshold detection algorithm. Onset is detected in all channels of the six events. The data and seizure onset times are shown in S2 Fig. Here, we average the recruitment times *t*_*n*_ in each channel over all six events to get the mean recruitment time ${\tilde{\tau }}_{n}=\langle t_{n}\rangle$ and cascade duration (the total mean recruitment time) $\text{max}\left({\tilde{\tau }}_{n}\right)-\text{min}\left({\tilde{\tau }}_{n}\right)$. The averaging of recruitment times is representative of the system’s dynamics as the in-vitro experimental set up is well controlled compared to the EEG recordings. The cascade duration is consistent for choices of *s* > 0.65 and *np* > 16000; see S3 Fig.

Fig 1 summarizes the collection and analysis of mouse mEC data. This figure shows the positioning of the silicone probe in the slice along the mEC, an example recording of an event, and the scaled mean recruitment times ${\hat{t}}_{n}={\tilde{\tau }}_{n}/\text{max}\left({\tilde{\tau }}_{n}\right)$ from all six events.

### Seizure onset pattern grouping

We refer to the patterns of dynamic activity captured by the EEG electrodes and detected by the threshold detection algorithm as *onset patterns*. We first consider a subset of 27 generalized seizures: 12 seizures clinically classified as generalized onset from one individual and 15 seizures clinically classified as focal onset from multiple individuals. The onset patterns for these 27 events are shown in S4 and S5 Figs; note one focal onset seizure was excluded from further analysis as seizure onset was detected in only 18 out of 19 channels.

Based on initial visual inspection of the detected onset patterns we propose three groups:

1. Fast domino: short total recruitment time and no large lags between detected activity in consecutive electrodes;
2. Slow domino: long total recruitment time and no large lags between detected activity in consecutive electrodes;
3. Multiple domino: long total recruitment time and one or more large lags that separate electrodes into subgroups for which the detected activity has similar recruitment times.

This grouping is inspired by the slow and fast domino effect detailed in [7, 9]. Both papers consider the escape of nodes from quiescent to oscillatory states in small motif networks. The fast domino effect is where all nodes escape in quick succession, almost simultaneously: this is our fast domino onset where electrodes are recruited in quick succession and the total recruitment time of all nodes is small. The slow domino effect is seen when all nodes on a network escape but it takes longer between consecutive nodes to escape giving a longer total escape time: this is our slow domino effect in which there are small lags between recruitment of electrodes but the total recruitment time for all electrodes is longer than the fast domino onset. The multiple-domino group is a novel class identified in this paper where subsets of electrodes escape in either a fast or slow domino effect, and there is one or more large lag in time between subsets. This gives the effect of multiple domino cascades that could only be observed in larger networks (> 3 nodes).

To quantitatively delineate between the groups we chose an initial threshold between fast and slow total recruitment time to be 0.5s and the threshold between long and short lags to be 0.4s. We then compute linear discriminants based on this data set using MATLAB. We use these delineations to assign each seizure, including the original 26, to one of the groups listed above.

**Fig 2:**
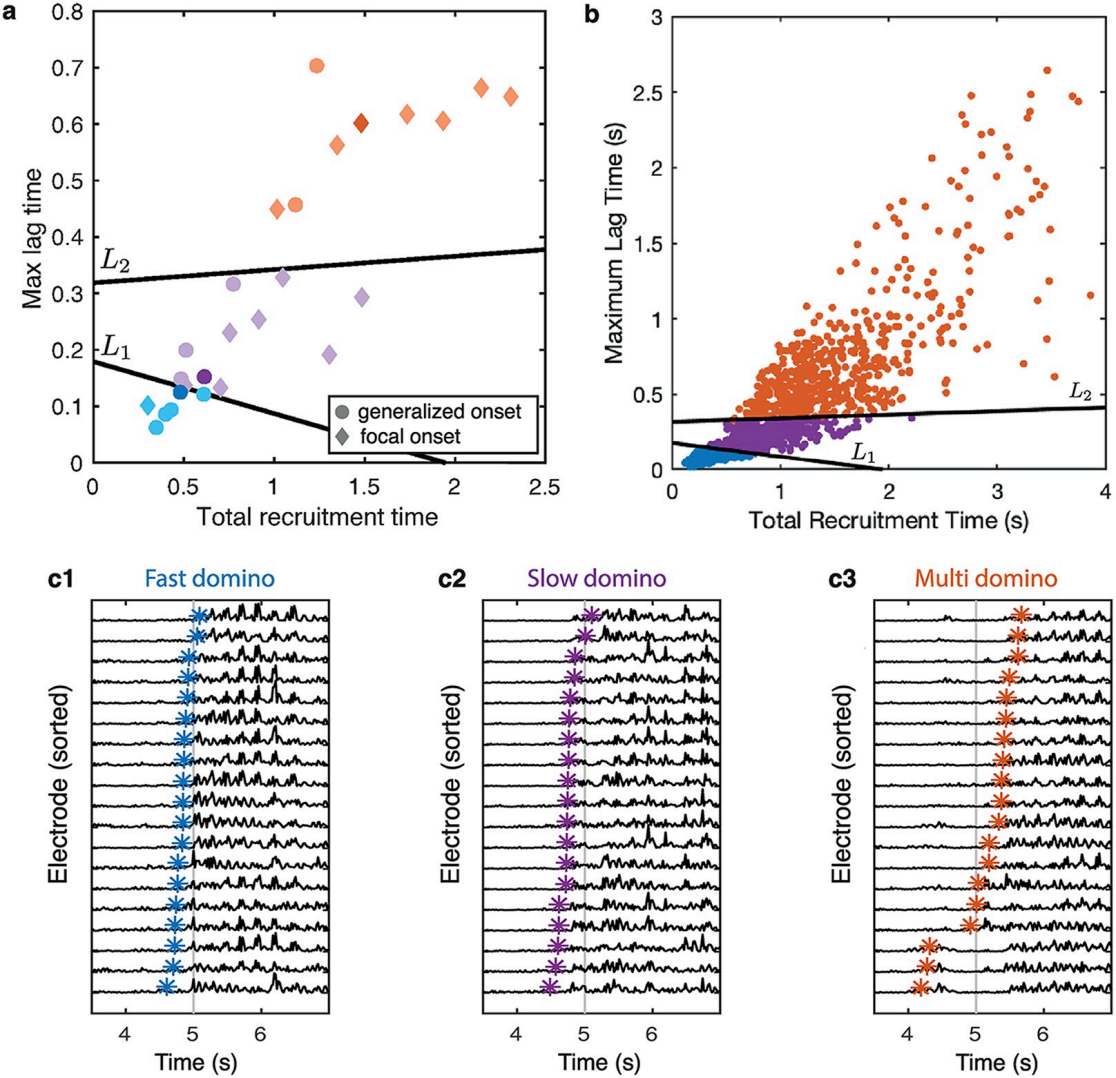
Total recruitment and maximum lag times in seconds (s) indicate a variety of seizure onset patterns. Panel **a** shows seizures from one individual, 12 clinically classified as generalized onset (•) and 3 classified as having focal onset (⧫); with an additional 11 seizures from multiple subjects classified as having focal onset (⧫), with the lines *L*_1_ and *L*_2_. One example seizure from each group, marked by dark blue, dark purple and dark orange in panel **a** are shown in **c1**–**c3** with the seizure onset time marked in each channel (*). Panel **b** shows the remaining 1145 seizures from 59 individuals with the lines *L*_1_ and *L*_2_ as in panel **a**.

We test how changing the parameters *np* and *s* of the seizure detection algorithm affects the group assignment of the 26 onset patterns from patient 1 and all focal events, described above. S6–S9 Figs show the recruitment and lag times for the three example seizure onset patterns detected using *np* = 60 fixed and *s* ∈ [0.4, 0.8] and using *s* = 0.60 and *np* ∈ [40, 80]. We compute the median recruitment and lag times for each event for both fixed values of *np* and *s*. Overall, the median time values for the three examples, namely fast, slow and multi-domino onset patterns, correspond to the same group assignment as the times computed for *np* = 60, *s* = 0.6 when varying either *np* or *s*. S9 Fig show the recruitment and lag times for all 26 events computed using *np* = 60, *s* = 0.6 (shown in Fig 2) and the median times for varying both *np* and *s*. Specifically, for fixed *s* = 0.6 the median times computed for *np* ∈ [40, 80] give the same group assignment as the times for *np* = 60, *s* = 0.6 in 81% of the 26 events. For fixed *np* = 60 the median times computed for *s* ∈ [0.4, 0.8] give the same group assignment as the times for *np* = 60, *s* = 0.6 in 96% of the events. The group assignment is robust to the choice of threshold modified by *s* in the detection algorithm, but is more sensitive to the envelope modified by *np*.

### Mathematical model

Coexistence of stable (attracting) background and seizure states has been implied to underlie mechanisms of transition to epileptic seizures in the context of both generalized and focal seizures [17, 18]. Bistability has been used effectively in phenomenological models of seizures driven by a desire to understand the underlying fundamental mechanisms of seizure transitions [8, 11].

We consider a network of coupled bistable nodes where each node represents one electrode on either the EEG placed on the scalp or the silicone probe array used in the mEC. The network of nodes is given by a system of stochastic differential equations

$$\begin{array}{c} dz_{n}\left(t\right)≔\left[f\left(z_{n};\nu_{n}\right)+\beta \sum_{m}A_{n,m}\left(z_{m}-z_{n}\right)\right]+\alpha dW_{n} \end{array}$$ (1)

for z∈C with *n* = 1, …, *N* [7, 9]. For the EEG data *N* = 19, the number of electrodes placed on the scalp, and for the mouse mEC data *N* = 16, the number of electrodes on the silicone probe.

The intrinsic bistable dynamics of each node is given by the following function,

$$\begin{array}{c} f\left(z;\nu \right)≔\left(-\nu +i\omega \right)z+{2z|z|}^{2}-z{\left|z\right|}^{4}. \end{array}$$ (2)

This is a truncated form of a Hopf normal form (or Bautin bifurcation) analyzed in [8, 9] based on [10]. Note here that we substitute *ν* = 1 − λ in the equation from [8]. We choose the parameter 0 < *ν* < 1 so that *f* admits two stable states; the seizure state represented by a periodic orbit with phase *ω* and the background state represented by a fixed point. The bifurcation diagram for (2) is shown in S10 Fig.

Each node starts in the background state (*z* = 0) and transitions to the seizure state are driven by noise when there is no coupling. Specifically, an independent identically distributed noise process *W*_*n*_ representing input from other neural or external stimuli is added to each node. The noise amplitude *α* is fixed throughout at *α* = 0.05 as in [7, 9]. Moreover, we assume that the time taken to transit back from seizure to background state is large enough to be ignored. When coupling is applied to the nodes, transitions to the seizure state are driven by both the noise and inputs from other nodes. S10 Fig illustrates the behavior of this model by showing a simulation of the network with *N* = 16 nodes.

The parameter *ν* controls how easily each node can be destabilized by noise; hence *ν* represents the *node excitability*. When *ν* is close to 0 small noise perturbations is sufficient to push the node into the seizure state; the node is excitable. Whereas when *ν* is close to 1 much larger noise perturbations are necessary for the node to transition; the node is more robust, i.e. less excitable. Each node is assigned a value of *ν*, these values are chosen as described in the Excitability section below. For simulations in which we fix excitability equal for all nodes we use *ν* = 0.15 in line with [7, 9] unless otherwise stated. Note that the transitions do not depend on the phase and so we fix *ω* = 0 for the simulations throughout unless otherwise stated. In this way we assume synchronization of nodes in the network and the model captures the observed phenomenon of phase locking at seizure onset.

The coupling between nodes is linear diffusive coupling, chosen in line with previous studies [7–9, 19, 20]. Such coupling could be seen as representative of local field potential mediated coupling via e. g. synaptic mechanisms at the level of the recordings obtained in the mouse mEC slice preparation and brain surface electrical potential coupling at the level of the EEG recordings. Nodes are coupled according to a weighted adjacency matrix *A* where entry *A*_*n*,*m*_ ≠ 0 if there is a connection from node *m* to node *n* and *A*_*n*,*n*_ = 0. The overall intensity of the coupling is governed by the scaling factor *β*; if *β* = 0 the nodes are disconnected. We fix the scaling factor *β* = 1 when modeling the mouse mEC data. When modeling the EEG data we vary *β* according to functional connectivity networks reconstruction based on individual seizure events and specify the values in the text. In the case of EEG modeling *A*_*n*,*m*_ is given by the association matrix computed for each seizure event as explained below.

#### Network structure

A plausible network structure between electrodes is required to apply the model to the EEG and mEC data. Linking mechanisms for neurons in a cohesive cortical network spanning the brain are not yet known [21].

For the EEG data, we use a standard measure of functional connectivity, namely a nonlinear regression method to compute the *association index* denoted $A_{n,m}\in \left(0,1\right)$ that assigns a functional connection strength from node *m* to node *n* [22, 23]. This association index represents a correlation ratio, i.e. the fraction of the variance of the time series *x*_*n*_ from node *n* that can be “explained” by the input time series *x*_*m*_ from node *m*. This method has been shown to be robust and applied to human intracerebral EEG data for characterizing seizure patterns [24–26]. Larger values of $A_{n,m}$ indicate a stronger functional connection between electrode activity and therefore between network nodes. We compute $A_{n,m}$ using the equation from [23]; detailed in S1 Text. The computations are implemented using a custom EEG-analysis toolbox [27] in Matlab and the association indexes for the three example seizures are shown in S11 Fig. We use each association matrix as the weighted adjacency matrix *A* in the model.

To test the dependence of $A_{n,m}$ on the time interval used, we first compute $A_{n,m}$ for 5s intervals, 0-5s, 5-10s and 10-15s. Next, we compute the Pearson dissimilarity as defined in [28] between pairs of functional connectivity (or association) matrices. We apply this by comparing the matrices computed for 5s intervals, 0-5s, 5-10s and 10-15s with $A_{n,m}$ computed using the whole interval; see S12 Fig. The functional connectivity matrix characterized by the smallest Pearson dissimilarity is found to be the 5-10s. Note that this is the interval that contains the seizure. Finally, the onset patterns detected using the threshold detection algorithm show that onset ranges from 4-6 s from the start of the epoch. Therefore, to ensure all onset information is captured for each seizure we use $A_{n,m}$ computed based on the whole 15s epoch.

For the mouse mEC data, we choose a network topology based on the geometry of the recording positions of the silicone probe in a comb-like structure (see Fig 1) that we represent mathematically by a one-dimensional chain of nearest neighbor coupled nodes. Due to the fact that the slice is thin, only 400 *μ*m, we ignore long range connections as they would be present (if any) with a low probability. Therefore, we assume a bidirectionally coupled chain where each node receives input from its neighbors *A*_*n*,*n*−1_ ≠ 0, *A*_*n*,*n*+1_ ≠ 0 and *A*_*n*,*m*_ = 0, if *m* ≠ *n* − 1, *m* ≠ *n* + 1. We also assume that the coupling weight between two consecutive nodes is the same in each direction, so *A*_*n*,*n*+1_ = *A*_*n*+1,*n*_.

#### Excitability estimation from EEG data

We estimate the value for the excitability parameter for each node in the model from EEG data by means of a measure of energy from the signal from each electrode as in [29]. The time-dependent energy is computed using a 1s sliding window with 50% overlap as

$$\begin{array}{c} E_{n}^{t}=\sum_{k=t/\delta }^{(t+1)/\delta }x_{n}^{2}\left(k\delta \right). \end{array}$$ (3)

Here *x*_*n*_(*kδ*) is the time series of node *n* at time *kδ* with *δ* as sampling time step. The sum is taken over the time interval [*t*, *t* + 1]. We then compute the total energy as

$$\begin{array}{c} E_{n}=E_{n}^{t_{1}}+E_{n}^{t_{1}+0.5}+\cdots +E_{n}^{t_{2}} \end{array}$$ (4)

In particular for the two chosen seizures in the EEG data, we use *t*_1_ = 0*s* and *t*_2_ = 14*s*, consistent with whole interval used for the association index calculation. To ensure reasonable computing time for model simulations and to normalize the energy profile range for all events, we scale this energy profile *E*_*n*_ to [0.1, 0.2]. The scaled energy profile is denoted by $E_{n}$ and these values are used to compute the node excitability as $\nu_{n}=0.3-E_{n}$ for each *n* which ranges between 0.1 and 0.2 unless otherwise stated. The energy profiles for the three examples are shown in S13 Fig.

#### Numerical simulations and comparison with data

We simulate the system given by Eqs (1) and (2) using a stochastic Heun method with time step *h* = 10^−3^ implemented in a custom code written in C++. We compute *K* ≥ 1000 realizations for each set of parameters. The initial condition is *z*_*n*_ = 0 for each node *n* = 1, …, *N*, where N is the total number of nodes in the network. Each realization has a different seed for the added noise processes. We detect the simulated seizure onset time *τ*_*n*_ for each node *n* as the first time a realization crosses the threshold *ξ* as

$$\begin{array}{c} \tau_{n}≔\text{min}\{t:|z_{n}\left(t\right)\left|>\xi \right\} \end{array}$$

where the threshold is fixed throughout at *ξ* = 0.5. We then compute the recruitment time *t*_*n*_ = *τ*_*n*_ − min(*τ*_*n*_) for each node. We average recruitment time over realizations *T*_*n*_ ≔ 〈t_*n*_〉. Note that the node with the lowest recruitment time min(*t*_*n*_) is not necessarily the same in each simulation and so $T_{\sigma_{1}}=\langle t_{\sigma_{1}}\rangle >0$, where *σ*_1_ denotes the node with the lowest simulated mean recruitment time.

To compare the simulation results and the data we scale the simulated times by their maximum mean time *t*_*n*_/max(*T*_*n*_) and denote the scaled mean recruitment time as ${\hat{T}}_{n}=T_{n}/\text{max}\left(T_{n}\right)$ so the largest simulated scaled mean recruitment time $\text{max}({\hat{T}}_{n})=1$. We scale the EEG and mEC data in the same way, by taking *t*_*n*_/max(*t*_*n*_) and ${\hat{\tau }}_{n}/\text{max}\left({\tilde{\tau }}_{n}\right)$ respectively.

To quantify the model fit to the EEG data and compare between model simulations we compute the distance *d* between the simulations and the data. To this end, we split the 1000 simulation into 20 groups, order the nodes according to the mean recruitment time and average over all 50 realizations in each group. We then compute the least square distance between each group and the data. We calculate the median distance *d* and 95% confidence intervals using a standard method from [30]. We use the Mann Whitney U test [31] with the null hypothesis that *d* values are samples from continuous distributions with equal medians.

For the mEC data, we average over all realizations and calculate the least square distance *d* between the scaled mean times from the data and model. For the mouse mEC data we also compare the proportion of seizures where seizure-like onset starts in the ventral nodes 1–8 or in the dorsal nodes 9–16. Note, the proportion *P*_*V*_ of activity starting in the ventral end has been already estimated in [13] for a larger number of samples (43) as *P*_*V*_ ≈ 0.86.

## Results

### Seizure onset patterns in human EEG

The results of the linear discriminant analysis outlined in Material and Methods identify the following boundaries between the three groups, fast-domino, slow-domino and multi-domino onset. The slow-fast dividing line is given by *L*_1_ = 2.9644 − 1.5236*r* − 16.5419*l* and the fast-multi dividing line is given by *L*_2_ = 31.0766 + 2.301*r* − 97.5312*l* where *r* is the recruitment time and *l* is the maximum lag time.

Fig 2 shows the total recruitment times and maximum lag times for all seizures with the linear boundaries *L*_1,2_. Panel a shows the subset of 26 data sets consisting of all focal events and all events from a single chosen individual with JAE. The division between the multi and fast-domino onset delineated by *L*_2_ is much clearer than between the slow and fast-domino onset groups given by *L*_1_. Panel b shows all remaining generalized seizures. These seizures have been assigned to each group using the delineating lines. This shows a continuum of points over the three groups, and the spread of recruitment and lag times in the multi domino group is much larger than for the fast-domino onset group. The example onset patterns shown in Fig 2c are three consecutive events from the chosen individual.

Each of the three groups, fast-domino, slow-domino and multi-domino onset, contain generalized seizures that are clinically classified as focal onset and generalized onset. This grouping goes beyond this clinical classification and so we wish to understand in more detail how these onset patterns arise. To this end, we apply our modeling framework to these three events.

#### Heterogeneous connectivity and excitability contribute differentially to different onset patterns

We build a network model for each seizure by constructing an all-to-all directed network of 19 nodes (one for each electrode) and explore the effect of changing the weighted adjacency matrix that defines the coupling, in our case the association matrix, and the intrinsic node excitability defined by the energy profile; see Materials and methods section for details.

We use the modeling framework to test the effect of different coupling and excitability in the model. Specifically, we perform three numerical experiments using: (Ex.1) homogeneous coupling strength given by the average of the association matrix elements *A*_*n*,*m*_ with heterogeneous excitability given by the energy profile $E_{n}$ (shown in S13 Fig); (Ex.2) homogeneous excitability *ν* = 0.15 and heterogeneous coupling given by *A*_*n*,*m*_ (shown in S11 Fig); (Ex.3) heterogeneous excitability and coupling. We simulate the model informed by the three seizures shown in Fig 2c for *β* = {0.0, 0.005, 0.015}. We compare the model output to the data using a least squares distance *d*, as described in Material and Methods.

**Fig 3:**
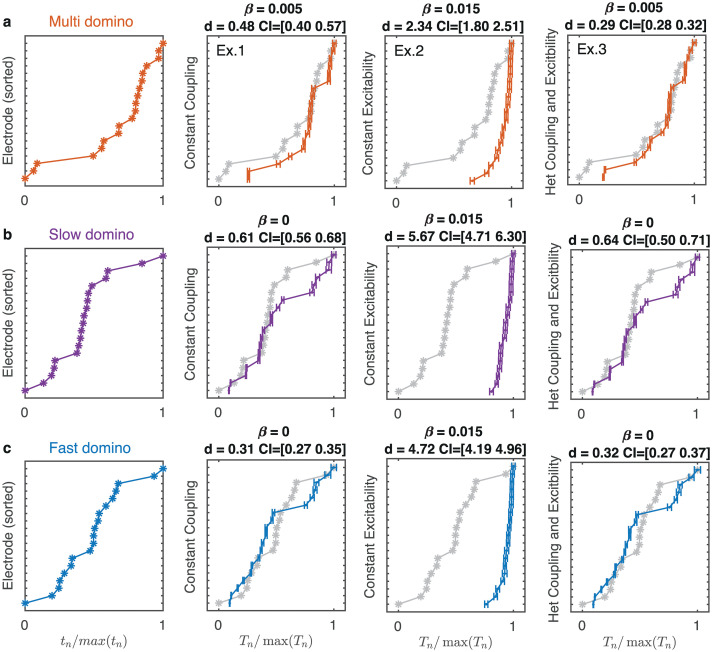
Simulated total mean recruitment times for the multi (a), slow (b) and fast-domino (c) onset patterns. The scaled recruitment times from the data are shown in column 1 for each of the three events; these are the same as in Fig 2c. These events are plotted as gray lines in the remaining panels, The scaled simulated total mean recruitment time *T*_*n*_/max(*T*_*n*_) and standard error bars for the numerical experiment with homogeneous coupling (Ex. 1, column 2), with homogeneous excitability (Ex. 2, column 3), and with heterogeneous coupling and excitability (Ex. 3, column 4). The simulations with lowest *d* value are shown with the corresponding *β* value and confidence intervals for *d* given in each panel.

Fig 3 shows the best (lowest *d*) model output for each experiment for each seizure. We note that the electrodes are not labelled here as here the focus is on recruitment and lag time properties, i.e. the dynamics of the system. All model simulations from the three numerical experiments for each pattern for *β* = [0.0, 0.005, 0.015] are shown in S14–S16 Figs.

In each set of simulations for (Ex.2) escapes happen almost simultaneously, as shown in Fig 3 column 3; see also S15 Fig. The large *d* values for this experiment for each seizure indicate that heterogeneous coupling alone cannot reproduce the variety of patterns observed.

For the multi-domino onset pattern the lowest *d*-values for Ex.1 and Ex.3 are for *β* = 0.005. The error for Ex.3 *d* = 0.29 is significantly smaller (p< 0.0001) than the error for Ex.1 *d* = 0.48. So we conclude that incorporating heterogeneous coupling and excitability in the model best captures the multi-domino onset pattern.

For the slow and fast domino the lowest *d* values for Ex.1 and Ex. 3 are for *β* = 0; *d* = 0.61 (Ex. 1) and *d* = 0.64 (Ex. 2) for slow domino, and *d* = 0.31 (Ex. 1) and *d* = 0.32 (Ex. 2) for fast domino. There is no significant difference between the *d*-values for Ex. 1 and Ex. 3 for either the slow domino onset (p = 0.78), or for the fast domino onset (p = 0.62). In this case the coupling weights are zero and the nodes escape independently governed by the excitability profile. This indicates that the excitability of nodes rather than the coupling is key to generating the fast and slow onset patterns.

To explore further the interactions observed in the *in vivo* data, we next apply our modeling framework to recordings of seizure-like neuronal activity from an *in vitro* experiment on mouse mEC.

### Seizure-like onset in mouse mEC

In this section, we demonstrate the applicability of our modeling framework to an experimental system at a different spatial and temporal scale. We consider the recruitment of seizure-like events in six experimental recordings from slices of mouse medial entorhinal cortex (mEC) that has been previously reported in [13] and is summarized in Fig 1. Panels **b** and **c** in Fig 1 clearly show a domino effect of the recruitment of nodes to the seizure-like state that takes around 20s to spread across all nodes over a distance around 1500*μ*m. We call this a slow-domino effect as it is much slower than the short interictal-like events propagated across the electrodes in the order of ms; see [13] Fig 3. We note that this domino recruitment is much slower than the total recruitment times for the seizures in the EEG recordings.

This *in vitro* system allows for some simplifying assumptions with regard to the network connectivity and excitability to be made. This in turn enables a more detailed exploration of the relative contributions of excitability and coupling weights in seizure onset patterns. To this end, we apply our network modeling framework to the mEC data and assess the contribution of excitability and coupling to the domino-like onset patterns, specifically the recruitment times and the proportion of ventral initiation.

To model the onset of seizure-like events in mEC, we build a network consisting of 16 nodes where each node represents one electrode of the silicon probe array; see Materials and methods section. We start with a simple case where coupling weights and excitability are homogeneous and fixed among the nodes; in particular we fix *ν*_*n*_ = 0.2, (*n* = 1, ⋯, 16). In this case, we find that the simulated mean recruitment times exhibit symmetry; see S17 Fig. Therefore, in the homogeneous system there is no discernible dorsoventral gradient due to network symmetry and the proportion of simulations with ventral initiation is *P*_*V*_ = 0.5. This indicates that homogeneous coupling weights and excitability cannot explain the sequential recruitment of seizure-like initiation along dorsoventral axis observed in the mouse mEC data. Thus, in the following subsections, we systematically explore how heterogeneous coupling or node excitability affect the domino-like recruitment along the chain.

#### Linear gradient in excitability facilitates slow-domino like sequential recruitment

Here we explore the effect of heterogeneous node excitability and constant coupling weight on recruitment times in the model. Experimental results have revealed the presence of functional dorsoventral gradients in a variety of excitability properties in neurons of the mEC [32]. In our modeling framework such experimental observations could be accounted for by changing the excitability parameter *ν* for each node linearly (in its simplest form). We note that this is the only parameter in the model that represents intrinsic properties of a node (in this case representing a small group of neurons in the mouse slice located in the vicinity of each of the silicon probes in the array). Accordingly, we define a linear gradient in *ν* given by

$$\begin{array}{c} \nu_{n}=\nu^{0}+\left(n-1\right)\delta \nu \end{array}$$ (5)

with *ν*^0^, *δν* > 0 for *n* = 1, ⋯, 16. Here we fix the coupling weights as follows, *A*_*n*,*n*+1_ = *A*_*n*,*n*−1_ = *A* = 0.1.

**Fig 4:**
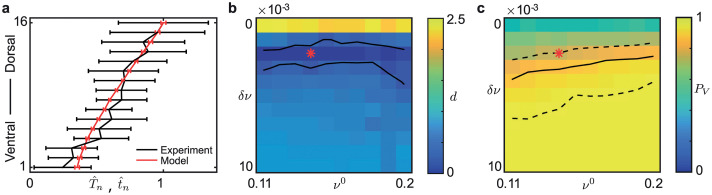
Modeling linear gradient in excitability with constant coupling *A* = 0.1. Panel **a** shows the scaled mean recruitment times from data, as in Fig 1c, and simulation results with gradient in node excitability given by (*ν*^0^, *δν*) = (0.14, 0.002) indicated by the red stars in panels **b–c**. Panel **b** shows the least squares distance *d* between the model and experimental data for different values of *ν*^0^ and *δν*; solid lines indicate *d* = 0.2. Panel **c** shows the proportion of ventral initiation *P*_*V*_; solid line is *P*_*V*_ = 0.86 and the dashed lines are the 95% confidence interval. The red star lies in the region of (*ν*^0^, *δν*) parameter space that both minimizes *d* and satisfies *P*_*V*_ ≈ 0.86.

Fig 4 shows the scaled mean recruitment times from the data ${\hat{t}}_{n}$ and the scaled mean simulation of the model ${\hat{T}}_{n}$ with the measure of difference *d* between the model simulations and the data and the proportion of ventral initiation *P*_*V*_ for different gradient values. In panels **b** and **c** the red stars indicate the parameter values used in the simulation of the model shown in panel **a**. Note that the electrode order in panel Fig 4a is the same as in Fig 1a.

When there is no gradient (*δν* = 0), the simulated results are in line with the findings using homogeneous excitability, *d* ≈ 2.5 and the proportion *P*_*V*_ = 0.5. The solid lines delineating *d* = 0.2 show a band 0.001 < *δν* < 0.003 across a range of *ν*^0^ where the difference between the model and the data is small. There is a region of overlap between this band and the region of (*ν*^0^, *δν*) contained within the dashed lines for *P*_*V*_. Fig 4b also shows that the gradient in excitability *δν* has a more significant effect compared to the initial level of excitability *ν*^0^ in capturing the observed sequential recruitment. Note that the value of coupling chosen here is important for recruitment speed. When using a lower value of the coupling weight *A* = 0.03, there is no overlap in the regions of *ν*^0^ and *δν* where *d* < 0.2 and within the confidence interval for *P*_*V*_ (see S18 Fig).

#### Modulation of excitability gradient controls recruitment speed

**Fig 5:**
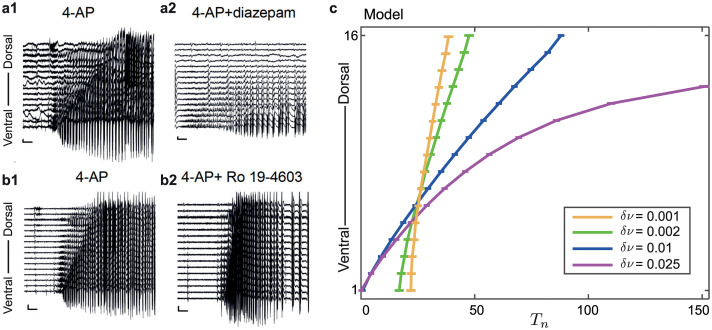
Modulating the excitability gradient changes domino speed. Example traces of seizure-like events induced with application of 4-AP in panels **a1** and **b1**, and with subsequent application of diazepam **a2** or Ro19-4603 **b2** [13]. Scale bar: 500 *μ*V, 5s. Panel **c** shows the mean recruitment time of model simulations of the network with different values of excitability gradient *δν*. Here we fix the coupling weight *A* = 0.1 and *ν*^0^ = 0.14 in line with the red star values in Fig 4. Increasing the gradient *δν* gives a slower-domino effect, so slow that the realization is terminated before all nodes escape, whereas decreasing the gradient gives as faster-domino effect.

Ridler *et al*. pharmacologically modulated the excitability levels in the slice [13] once the seizure-like activity was established with 4-aminopyridine (4-AP) by additional bath application of diazepam or Ro19-4603 (both GABA action modulators). It was reported that Diazepam decreased the speed of recruitment whereas Ro19-4603 increased the recruitment speed when compared to baseline (4-AP only). Using our modeling framework we are able to account for these experimental observations as shown in Fig 5a and 5b. Specifically in order to reproduce the chemically induced changes in recruitment speed it is sufficient to vary the excitability gradient *δν* in the model. For positive modulation we find that increase in *δν* suffice, resulting in a shallow excitability gradient and large difference in excitability between neighboring nodes; hence slowing down the domino effect. For negative modulation we find that decrease in *δν* is enough to produce a steep excitability gradient and small difference in excitability between neighboring nodes; hence speeding up the domino effect.

Fig 5c shows the mean recruitment time (not scaled) of the model simulations for different values of *δν*. Note that the electrode order in panel **c** is the same as in Fig 1a. As above we fix the coupling weight *A* = 0.1; we note qualitatively similar results were found for lower values of *A*. We take *δν* = 0.002 as the pre-modulation (4-AP only) value, as in Fig 4. When the gradient is much larger, *δν* = 0.025, the onset pattern has a very slow-domino effect and the total recruitment time is very large; here, due to the large recruitment times the realization is terminated before all nodes escape. This pattern of recruitment captures the very slow-domino onset pattern in panel **a2** modulated by 4-AP + diazepam. When the gradient is decreased, *δν* = 0.001 the total recruitment time is small and the domino-effect is much faster, suggesting more synchronized recruitment. This captures the fast-domino onset shown in panel **b2** modulated by 4-AP + Ro 19-4603. For *δν* = 0.001 the mean recruitment time for node 1 is larger than that for other values of *δν* and is far from 0 because when simulated node 1 is not always the first node to initiate the recruitment.

#### Linear gradient in coupling delineates regimes of slow and fast-domino recruitment

Next we proceed to explore the effect on recruitment times using heterogeneous coupling and constant excitability *ν* = 0.2 for all nodes in the model. It has been experimentally suggested that the dorsal mEC receives a greater number of inhibitory inputs than the ventral mEC [13, 33] and is hence more resistant to transitions between different dynamic states. As above we look for the simplest possible way to account for these observations in our modeling framework. One way to do this is by assuming heterogeneous dorsoventral coupling weights. To this end, we incorporate a simple but general linear gradient in coupling weights along the chain-like network structure. Specifically, we use the weighted adjacency matrix

$$\begin{array}{c} A_{n,n+1}=A_{n+1,n}=A^{0}+\left(n-1\right)\delta A \end{array}$$ (6)

where *δA*, *A*^0^ > 0 for *n* = 1, …, 16. We note that in this case the coupling weights increase from the weakest between nodes 1 and 2 (ventral—fewer inhibitory connections) to the strongest between nodes 15 and 16 (dorsal—more inhibitory connections).

**Fig 6:**
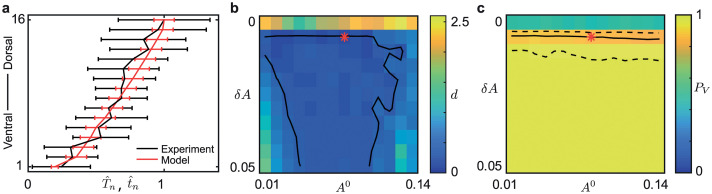
Modeling linear gradient in coupling with constant excitability *ν*_*n*_ = 0.2 for all nodes. Panel **a** shows the scaled mean recruitment times ${\hat{t}}_{n}$ extracted from the experimental data, with the simulation results with gradient in coupling weights (*A*^0^, *δA*) = (0.08, 0.005) indicated by the red stars in **b** and **c**; compare to Fig 4. The red stars lie in the region of (*A*^0^, *δA*) parameter space that both minimizes *d* and satisfies *P*_*V*_ ≈ 0.86.

Fig 6a depicts the scaled mean recruitment times ${\hat{T}}_{n}$ from the data and the model simulations for parameter values (*A*^0^, *δA*) = (0.08, 0.005). Note that the electrode order here is the same as in Fig 1a. The difference *d* between the model and the data, and the proportion *P*_*V*_ of realizations initiated in the ventral end of the network are also shown for a range of coupling gradient values; compare to Fig 4. When there is no gradient, *δA* = 0, *d* ≈ 1 and the proportion *P*_*V*_ = 0.5 is in line with simulated results using homogeneous coupling. In panel **b**, the lines at *d* = 0.2 delineate the different coupling regimes identified in [9]. If *A*^0^ small (*A*^0^ < 0.02) then the coupling is weak and the network recruits as if uncoupled. For strong coupling *A*^0^ is large, the recruitment of nodes becomes more synchronized (fast domino effect) and *d* is large (*d* > 0.2). The region with lowest difference to the data (*d* < 0.2) is within the intermediate coupling regime, between the solid lines, in which the slow domino effect can be seen. Panel **c** shows that for *δA* ≠ 0 the proportion *P*_*V*_ ≈ 1. We find values (e.g. indicated by the red star) of *A*^0^ and *δA* where *d* < 0.2 and *P*_*V*_ ≈ 0.86 in line with [13].

This shows that the network model with a linear gradient in coupling weights delineates regions of slow and fast domino-like recruitment. Moreover, this model captures the behavior of recruitment times seen in the experimental data which is well explained by the slow-domino effect.

#### Severed coupling facilitates time lag in recruitment

Finally we test how modulation in coupling affects recruitment patterns. To this end, we consider the anatomical separation of the dorsal and ventral portions of the mEC slice via an incision in the intermediate mEC with a scalpel blade, as reported in [13]. Seizure-like activity was found to initiate in the ventral mEC before the dorsal mEC, but transitions in the dorsal mEC took longer to initiate compared to intact slices. This suggests that the dorsal mEC is less likely to produce seizure-like activity in the absence of the ventral mEC. We model this scenario and predict the mean recruitment times along the 16 nodes in the chain. Specifically, we model the anatomical separation by setting *A*_8,9_ = *A*_9,8_ = 0. Moreover, we fix the coupling weights

$$A_{n,n+1}=A_{n+1,n}=A>0$$

for *n* ≠ 8 and fix the gradient in excitability (*ν*^0^, *δν*) = (0.14, 0.002) as in Fig 4.

**Fig 7:**
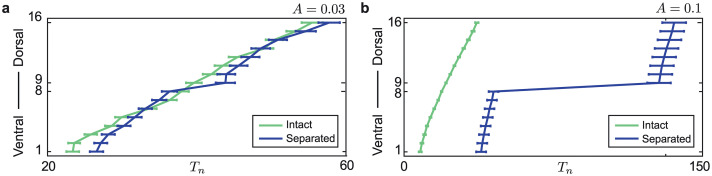
Separation of dorsal and ventral nodes produces a multi-domino effect. Panels **a** and **b** show simulated mean recruitment times for both intact (green) and separated (blue) chain structures with indicated coupling weights *A*. In both cases the separated chain structure shows a lag between nodes 8 and 9. Panel **a** shows that when the coupling strength is weak the separated simulation has a slow-domino or multi-domino onset with one larger lag than the intact onset, whereas in panel (b), *A* = 0.1, the lag is larger and two domino cascades clearly form.

Fig 7 shows the mean recruitment times from model simulations for intact and separated coupling structures with either weak coupling *A* = 0.03 or intermediate coupling *A* = 0.1. When the chain is severed, recruitment is disrupted. The recruitment times form two clusters (the ventral cluster with nodes 1-8 and the dorsal cluster with nodes 9-16) separated by a lag in recruitment time. When the coupling is weak *A* = 0.03 the lag is larger than the intact but could still be considered as a slow-domino effect. However, the size of the lag is more pronounced when the coupling weight is intermediate *A* = 0.1 and the onset pattern is clearly multi-domino with two cascades. This result is in line with experimental findings where the dorsal mEC takes a long time to transit to seizure-like state in the absence of influence from the ventral mEC. This adds further support for the use of intermediate coupling weights in the model.

## Discussion

The variety of generalized seizure onset patterns observed in clinical EEG recordings have not been fully explored. EEG is the dominant tool used by clinicians and neurologists to diagnose epilepsy therefore analysis and modeling of the complex patterns observed may provide crucial insight for diagnosis and treatment of this complex disorder [34].

Using an exemplary data set of recordings from individuals with idiopathic generalized epilepsy (IGE) [4, 12] we show a variety of spatiotemporal onset patterns for seizure in the data that can be characterized by the total onset (recruitment) time and the lags in onset time between electrodes. Here we use these two features to suggest a novel taxonomy consisting of three types of patterns: fast-domino onset where activity is detected almost simultaneously by all electrodes; slow-domino onset where the onset of activity takes longer to be detected in all electrodes; and multi-domino onset where several domino-like cascades of activity are observed across groups of electrodes. These novel categories transcend the existing classification of generalized or focal onset of a generalized seizure.

We develop and apply a network modeling framework of seizure onset to capture different seizure onset patterns characterized by the total recruitment time and lags between electrodes. We demonstrate, as a proof of concept, that this novel framework can be applied at different scales and used to explore the contribution of node level properties on emergent network behavior.

To this end, we apply this network model to qualitatively capture three seizure onset patterns, one from each onset pattern category. The illustrative examples used here are taken from one individual diagnosed with Juvenile Absence Epilepsy (JAE, the most common syndrome within the data) [4, 12]. The precision approach taken here by modeling individual seizures is in line with contemporary approaches to epilepsy modeling based on inference of the network structure and node excitability (propensity to transition) from the data for each seizure [19, 20]. Our modeling results show that heterogeneous node properties and heterogeneous coupling weights play non-trivial roles in the formation of the different onset patterns. We show that, of the two heterogeneities, heterogeneous excitability plays a more dominant role in shaping the emergent behavior for all the three onset patterns. Indeed, heterogeneous excitability alone is sufficient to reproduce the fast and slow-domino onset patterns. The multi-domino onset pattern is best captured by incorporating both heterogeneous coupling and excitability, where the heterogeneous coupling provides a refinement to the multi-domino onset pattern.

In order to further investigate the contribution from node excitability heterogeneity or coupling heterogeneity separately, and to demonstrate that the framework can be used at the micro scale, we apply the network model to multi-electrode array recordings from mouse mEC [13]. The mEC data shows an induced domino-like seizure recruitment from the ventral to dorsal mEC. To model this, a chain network structure is assumed based on experimental set up. The effects of gradients in node excitability or coupling weights are explored based on model simulations. We capture experimental findings by showing that steeper gradient in excitability leads to fast-domino onset patterns where nodes are recruited in quick succession. Moreover, we show that shallower gradient in excitability leads to slow-domino onset patterns where for very steep gradients, long recruitment times are found and can cause the propagation to effectively stop. We also show that a discontinuity in the coupling leads to a time lag in recruitment in the chain, creating a multi-domino onset effect; these predicted recruitment patterns could be experimentally tested in the future.

The idea of groups of electrodes which detect similar changes in activity at seizure onset or termination was explored by Proix *et al*. for focal seizures [35]. The authors considered the spatiotemporal patterns of seizure recruitment and termination detected by stereotactic EEG. They identified that although some seizures begin and terminate synchronously across electrodes, others form distinct clusters where nodes within each cluster are recruited or terminate (almost) simultaneously, with a significant time lag between each cluster. They focused on termination patterns and use a neural field model based on an extension to the so-called “Epileptor” model [36] to show that recruitment correlates to weak (structural) connections. This paper extends the concept to the onset of generalized seizures and our finding that coupling plays an important role in the multi-domino onset pattern is in line with their findings.

Network topology alone is a necessary but not sufficient condition for generalized seizures to emerge. For example, Chowdhury *et al*. demonstrated that alterations in brain network topology were present in both people with idiopathic generalized epilepsy, as well as the unaffected first-degree relatives in comparison to healthy controls [37]. Moreover, Petkov *et al*. demonstrate increased mean degree is related to seizure onset in generalized seizures [38]. Consequently, Terry *et al*. studied a phenomenological model of seizure onset in which each node could transition between background and seizure states [11]. They found that increasing the excitability of a single node in a motif (four-node) network could produce dynamics consistent with either focal, generalized or focal to bilateral ictal activity. In that model, the long term behavior of the system (how many nodes are in the ictal state) was considered. Building on these initial mathematical descriptions, Schmidt *et al*. extended this framework to consider a modular network of tightly coupled Kuramoto type oscillators representing activity within each node, and a functional network between nodes informed by clinical EEG [39]. Therein, onset patterns either defined by cycles within the functional network or synchronous behavior within specific nodes were described. In the context of generalized epilepsy, this approach was shown by Schmidt *et al*. to have predictive value for revealing epilepsy from epochs of EEG data that would be considered clinically negative (e.g. free from discharges or other abnormalities) [40]. Very recently, this approach has been further extended by Woldman *et al*. to account for different network properties characterizing both focal and generalized seizures [41].

Martinet *et al*. show that network characteristics can influence recruitment dynamics of the neocortex in focal to bilateral tonic-clonic seizures [42]. In particular, recruitment times and the degree of spatial organization during onset have been shown to be patient specific and large variations exist between patients. More recently a cascading failure model was used to simulate the neural networks underlying generalized tonic-clonic seizure [43]. They use functional networks derived from graph theory and initiated seizures via stimulation of the node with the largest number of connections. They do not explore the effect of inherent excitability of the nodes to seizure initiation.

In this framework we use a standard measure of functional connectivity from the EEG to inform our coupling structure and weights. Due to the ambulatory nature of the EEG recordings we chose an all to all connected network structure. For each of the two seizure events studied in detail here we computed and compared the functional connectivity matrices obtained from different sections of the event epoch as well as by using the entire epoch. The differences between matrices computed from within one epoch were found to be smaller than the difference between the two epochs. Therefore we chose to use the matrix computed from the entire epoch in each case. There is evidence that functional connectivity changes with time, particularly before and during epileptic seizure [44–46]. A future development of the model could be to include a dynamic coupling structure to explore the role of evolving connectivity in conjunction with excitability on seizure onset patterns including multiple seizure events and on a longer time scale. Furthermore, cortical excitability is known to change before, during and after a seizure [1]. A further development of the model could be to include a dynamic excitability on the nodes. This could be incorporated by making *ν* a slow variable on the order of minutes or hours compared to the total recruitment time of the seizure onset on the order of milliseconds.

A number of computational and mathematical models have explored the influence of intrinsic node properties on the dynamics of recruitment to a pathological state; see for example [47–49]. Notably, Goodfellow *e*t al. showed that spatial heterogeneity is involved in the onset of absence seizures [50]. Building on the computation models of spike-wave discharges observed in EEG during absence seizures by [51] and further extended by [52, 53], Goodfellow *et al*. develop a spatial extended Jansen-Rit model and show that seizure onset may relate to the influence of local heterogeneous excitability. However, they do not explore how the interplay between connectivity and heterogeneous excitability produce different seizure onset patterns.

There are multitude approaches to elucidating brain region excitability measures from neuroimaging data. Most are applied in the problem of epilepsy surgery for Focal epilepsies (rather than generalized epilepsies considered here). The first and second generation Epileptor models have been used to model and predict seizure recruitment of local and distant brain regions in focal epilepsy [20, 36, 54, 55]. The authors characterized regimes of recruitment behavior for different coupling weights [55] but do not consider excitability gradients or recruitment timings. Jirsa et al. take two approaches to identify a brain excitability map for their Virtual Epileptic Patient described in [20]. Having constructed a personalized Epileptor network model using structural connectivity measures from diffusor tensor imaging (DTI), they apply heterogeneous excitability to the nodes first as prescribed by an expert clinician then using excitability elucidated form functional stereotactic EEG data using Bayesian inference methods. Using the first they are able to simulate seizure patterns observed from the patient. With the second they use the excitability measure itself to identify the seizure onset zone to inform surgery for resection. Another approach to approximate excitability was taken by Hutchings et al [56]. The authors elucidate heterogeneous measures of excitability and connectivity using DTI data from individual patients with temporal lobe epilepsy. They then apply a Bautin bifurcation model based on [8] to simulate surgery (resection of nodes) and predict outcome success.

Although our model contains oscillatory (seizure) states it does not take into consideration, for example, the frequency of oscillations as all nodes are synchronized by design. The results presented here do not depend on the choice of node model given by (2). We note that we repeated the model simulations in the mouse mEC section using a simple bistable model with two steady states (rather than one oscillatory) on each node and the results are consistent. Several other mathematical models have been designed to reproduce the activity and transitions between resting and seizure states, including neural mass models [19, 48, 54]. These could be used to incorporate different levels of biophysical reality. Moreover, a generalized Hopf model that incorporates additional phase terms with phase dependent coupling could be used to explore the effect of synchronization on onset patterns. We consider these beyond the scope of this paper.

A future application of this framework would be to model the non-seizure events that have been observed in resting state EEG recordings and are also generated in mouse mEC experiments [13]. Expanding the framework to investigate the initiation, recruitment and recurrence of inter-ictal spikes may provide further insight into the structural and functional alterations indicative of different types of seizure and inter-seizure events. This expansion would rely on using suitable intrinsic node dynamics in place of Eq (2). The question of which node dynamics to impose relies on making assumptions regarding the mechanism that defines the transition from background activity to a seizure or inter-ictal spikes; see for example the comparison of the choice of node dynamic models on outcome in the context of epilepsy surgery [49].

To assign seizure onset patterns to the fast, slow, and multi-domino onset groups we use linear discriminant analysis. We show that this assignment is robust to choices of the parameters of the seizure onset detection algorithm in S6–S9 Figs and note that the median over a range of parameters could be used as an alternative to the fixed choice of parameters used here. The lines delineating the groups are based on an initial assignment of points by visual inspection (see Materials and methods). The onset patterns could instead be submitted to a machine learning algorithm to formulate a classification. In this study, for consistency with the other measures, we use the entire EEG epoch of the data provided to extract seizure onset pattern. A smaller interval around the seizure onset as identified by the clinician could be used to give a clearer grouping; for example S19 Fig shows the results of submitting the first 8 seconds of each EEG epoch to the seizure detection and linear discriminant analysis. Whilst for the chosen subset of events the groups are somewhat more clearly distinguished, S19 Fig also shows the spread of the generalized onset patterns over all three groups is maintained. The focus of this paper is to highlight the various clinically observed onset patterns and apply a modeling framework to identify key components to how they are generated. An important step towards implementation of this classification in clinical practice would require verification by future studies and identifying connections between the groups and, for example, treatment outcome.

S9 Fig shows that the multi-domino class is actually the most robust, as the median lies in the same classification as the point for *np* = 60, *s* = 0.6 for all multi-domino onset events. This may be due to the fact that the multi-domino onset region is the largest of the three in the recruitment/lag plane. We show that the exact order of electrodes (based on detected activity) varies with the parameters of our detection algorithm, but our novel assignment of domino-onset pattern is robust to these perturbations; see S6–S8 Figs.

We are using 19 electrode EEG, which is unsuitable for source localization techniques and so we cannot make conclusions about the excitability of underlying brain regions nor place significance on the precise order of electrodes here. Future work would be to incorporate high density or intracranial EEG recordings to link the electrode order in the domino cascades to the underlying generators of epileptic activity. In particular, we observe for the multi-domino onset example shown in S8 Fig that for the onset patterns classified as multi-domino onset the maximum lag always precedes recruitment of electrode C4. We note that the association matrix shown in S11 Fig, electrode C4 has lower connectivity values than other nodes for the multi-domino event. This is incorporated in our modeling results shown in Fig 3 where row a column 4 shows the best fit for the multi-domino case where the largest simulated lag proceeds electrode C4 (not labelled). This raises the question of how the persistence of this lag relates to the underlying neurodynamics.

We present here, a variety of clinically observed onset patterns and a modeling framework applied at multiple scales to show that the interplay between excitability and coupling is required to generate them. Together these constitute a step toward a more comprehensive understanding of different types of seizures and therefore a more advanced classification of the epilepsies.

## Supporting information

S1 Text Association index. (PDF)

S1 Fig Visualization of the steps for the threshold detection algorithm applied to one channel. Panel **a** shows the raw data from one channel from an EEG recording. Panel **b** shows the data, band pass filtered to 4-20Hz, z-normalized and rectified. An envelope shown in blue is computed using spline interpolation over local maxima separated by at least *np* = 60 data points. Seizure onset time is the first time over a threshold defined by *s*×standard deviation from the mean of the envelope time series, here *s* = 0.6 for the EEG data, shown in panel **c**. This process is repeated for all channels in each epoch. The values of *np* and *s* are adjusted for the mEC data appropriately and tested for robustness in S3 Fig.
(EPS)

S2 Fig The six mEC recordings, labelled a–f, described in Section Mouse mEC recording. The results of applying the threshold detection algorithm to each channel are marked with a red star. The average of the red stars in each channel is shown in Fig 3c. Electrode 1 is at the ventral end of the slice, electrode 16 is at the dorsal end of the slice; this is fixed throughout.
(EPS)

S3 FigRobustness in extracting recruitment times when altering parameters *np* and *s* in the algorithm for mEC data.Panel **a** shows cascade duration (the change of mean recruitment time ${\text{max}}_{n}\left({\tilde{\tau }}_{n}\right)-{\text{min}}_{n}\left({\tilde{\tau }}_{n}\right)$) for the mEC data when altering *np* or *s*; red curves are for fixed *s* = 1 and varying *np* while black curves are for fixed *np* = 22000 and varying *s*. Panel **b** shows the mean recruitment times for various *s* with fixed *np* = 22000 (top) and for various *np* with fixed *s* = 1 (bottom). Note that the order of channels from ventral to dorsal is the same in each panel.(EPS)

S4 Fig The seizure onset patterns of the 15 generalized seizures clinically classified as focal onset. The stars mark the seizure onset time for each electrode. In panel **c** seizure onset is only detected in 18 of the 19 channels and this seizure is excluded from further analysis. Panel **d** shows the original assignment of onset patterns, where the separatricies are at total recruitment time = 0.5 and maximum lag = 0.4. Panel **a** shows onset patterns that were assigned multi-domino onset, panel **b** shows the onset pattern assigned fast-domino onset, and panel **e** shows onset patterns assigned slow-domino onset.
(EPS)

S5 Fig Variety of seizure onset patterns from one subject with JAE. 12 generalized seizures are classified as generalized onset, three are clinically classified as focal onset. The stars mark the seizure onset time for each electrode. Panel **d** shows the original assignment of onset patterns, where the separatricies are at total recruitment time = 0.5 and maximum lag = 0.4. Panel **a** shows onset patterns that were assigned multi-domino onset, panel **b** shows the onset pattern assigned slow-domino onset, and panel **e** shows onset patterns assigned fast-domino onset.
(EPS)

S6 Fig Robustness for the fast-domino seizure onset pattern when altering parameters *np* and *s* in the seizure detection algorithm. The top row shows the recruitment and lag times for the seizure onset patterns detected using *np* = 60 fixed and 0.4 < *s* < 0.8. Seizure onset patterns are shown for *s* = 0.4, 0.6, 0.8. The bottom row shows the recruitment and lag times for the seizure onset patterns detected using *s* = 0.60 fixed and 40 < *np* < 80. Seizure onset patterns are shown for *np* = 40, 60, 80. In the first column the linear classification lines *L*1 and *L*2 are marked. The point corresponding to *np* = 60, *s* = 0.6 is marked with a *, and the median over all points is marked with a +. For *s* < 0.54 the recruitment time and lag time become larger and the onset falls into the slow-domino onset group, however the median overall remains in the fast-domino group. For all values of *np* the onset is in the fast-domino onset group. Note that the electrode order in the onset patterns shown in the remaining panels changes for some values of *np* and *s*.
(EPS)

S7 Fig Robustness for the slow-domino seizure onset pattern when altering parameters *np* and *s* in the seizure detection algorithm. The top row shows the recruitment and lag times for the seizure onset patterns detected using *np* = 60 fixed and 0.4 < *s* < 0.8. Seizure onset patterns are shown for *s* = 0.4, 0.6, 0.8. The bottom row shows the recruitment and lag times for the seizure onset patterns detected using *s* = 0.60 and 40 < *np* < 80. Seizure onset patterns are shown for *np* = 40, 60, 80. In the first column the linear classification lines *L*1 and *L*2 are marked. The point corresponding to *np* = 60, *s* = 0.6 is marked with a *, and the median over all points is marked with a +. For 0.61 < *s* < 0.68 the lag time are large and onset falls into the multi-domino onset group, however the median remains in the slow-domino group. Varying *np* gives a spread of points over all three groups, but the median is in the slow-domino onset group. Note that the electrode order in the onset patterns shown in the remaining panels changes for some values of *np* and *s*, but seizure onset is first detected in electrode C3 in each case.
(EPS)

S8 Fig Robustness for the multi-domino seizure onset pattern when altering parameters *np* and *s* in the seizure detection algorithm. The top row shows the recruitment and lag times for the seizure onset patterns detected using *np* = 60 fixed and 0.4 < *s* < 0.8. Seizure onset patterns are shown for *s* = 0.4, 0.6, 0.8. The bottom row shows the recruitment and lag times for the seizure onset patterns detected using *s* = 0.60 and 40 < *np* < 80. Seizure onset patterns are shown for *np* = 40, 60, 80. In the first column the linear classification lines *L*1 and *L*2 are marked. The point corresponding to *np* = 60, *s* = 0.6 is marked with a *, and the median over all points is marked with a +. For all values of *s* the onset falls into the multi-domino onset group. For *np* < 77 the onset is classed as multi-domino onset, and the median is within this group. For larger *np* values the onset is classed as slow-domino. Note that the first three electrodes remain the same when the onset is classified as multi-domino, and that the maximum lag is always before node C4. The association matrix in Fig. S8. shows that C4 has low connectivity values.
(EPS)

S9 Fig Robustness of the classification of the points from a single patient and all the clinically classified focal-onset events to the detection algorithm parameters *np* and *s*. The first panel shows the classification of the 26 points points from a single patient with all the focal onset events for *np* = 60 and *s* = 0.6. The fast, slow and multi-domino example are in darker blue, purple and orange, respectively, with *L*1 and *L*2 marked; this is the same as Fig 1a but here is plotted on a log scale. The other two panels show these 26 points each with a corresponding median value (+) that indicate the results of varying *np* for *s* = 0.6, and varying *s* for *np* = 60. For fixed *s* = 0.6 the median values computed for 40 < *np* < 80 give the same classification as the points *np* = 60, *s* = 0.6 in 81% of the 26 events. For fixed *np* = 60 the median values computed for 0.4 < *s* < 0.8 give the same classification in 96% of the events. The algorithm is very robust to the choice of threshold modified by *s* but is more sensitive to the spline interpolation modified by *np*. We note that the multi-domino group is robust to all values of both *np* and *s*.
(EPS)

S10 Fig Panel a shows the bifurcation diagram of Eq (2) for fixed *ω* = 20. For *ν* < 0 there is one unstable equilibrium and a stable limit cycle. At *ν* = 0 there is a subcritical Hopf bifurcation that gives rise to an unstable limit cycle and stabilizes the equilibrium. For 0 < *ν* < 1 the system is bistable. At *ν* = 1 there is a saddle node of limit cycles and for *ν* > 1 there is one stable equilibrium. We restrict our *ν* values to the bistable regime where the stable equilibrium represents the background state and the stable limit cycle represents the seizure state. Panel **b** shows an example of node trajectories from the network model given by (1) with (2) for *N* = 16 nodes. Electrodes transition from resting (steady) state to seizure (oscillatory) state is driven by noise. Here we use coupling strength of *β* = 0.03.
(EPS)

S11 FigThe association index matrices A computed using Eq (1) above, for of the three example seizures shown in Fig 1a–1c.The association index matrices are not symmetric along the diagonal. The association index here is computed using the entire 15s epoch containing each seizure. The $A_{n,m}$ values shown here are used as the coupling weights *A*_*n*,*m*_ in the network modeling framework for each seizure.(EPS)

S12 Fig The association index computed for 5s intervals, 0-5s, 5-10s and 10-15s for each of the three example events. The Pearson dissimilarity [28] to the matrix using whole 15s interval shown in S11 Fig in comparison to matrix using [0-5], [5-10], and [10-15] intervals are respectively: for the fast-domino onset 0.1309, 0.0071, 0.1061; for the sow-domino onset 0.0952, 0.0022, 0.1439; and for the multi-domino onset 0.2166, 0.0026, 0.0835. In each case the interval [5-10] is has the smallest distance to the whole interval matrix.
(EPS)

S13 FigEnergy profiles of the three chosen examples shown in Fig 2 calculated using (3) and (4).The time-dependent energy $E_{n}^{t}$ calculated from the whole recording are shown with the scaled total energy profiles $E_{n}$. These energy profiles are used for the node excitability in the network model for each event. See ‘Materials and Methods’ for further details.(EPS)

S14 Fig Experiment 1: Model simulations computed with excitability given by the energy profiles shown in S13 Fig and homogeneous (constant) coupling given by the mean of the association matrix *A*_*n*,*m*_. The scaled recruitment times *t*_*n*_ from the data are shown in column 1 for the multi, slow and fast-domino onset pattern; compare with Fig 1c. These events are plotted as grey lines in the remaining panels. The scaled simulated total mean recruitment times *T*_*n*_ with standard error bars for *β* = 0.0, 0.005, 0.015 are shown in columns 2-4. The least squares distance *d* and 95% confidence intervals are given for each simulation.
(EPS)

S15 Fig Experiment 2: Model simulations computed with coupling given by the association matrix shown in S11 Fig and with equal excitability *v* = 0.15 on each node. The scaled recruitment times *t*_*n*_ from the data are shown in column 1 for the multi, slow and fast-domino onset pattern; compare with Fig 1c. These events are plotted as grey lines in the remaining panels. The scaled simulated total mean recruitment times *T*_*n*_ with standard error bars for *β* = 0.0, 0.005, 0.015 are shown in columns 2-4. The least squares distance *d* and 95% confidence intervals are given for each simulation.
(EPS)

S16 Fig Experiment 3: Model simulations computed with coupling given by the association matrix shown in S11 Fig and excitability given by the energy profiles shown in S13 Fig. The scaled recruitment times *t*_*n*_ from the data are shown in column 1 for the multi, slow and fast-domino onset pattern; compare with Fig 1c. These events are plotted as grey lines in the remaining panels. The scaled simulated total mean recruitment times *T*_*n*_ with standard error bars for *β* = 0.0, 0.005, 0.015 are shown in columns 2-4. The least squares distance *d* and 95% confidence intervals are given for each simulation.
(EPS)

S17 Fig Homogeneous excitability and coupling for modeling mouse mEC. Each panel shows the mean recruitment times from 2000 simulations with *ν* = 0.2 fixed for different values of *A*. There is no discernible dorso-ventral gradient or vice versa for any value of *A*. For weak and strong coupling, the recruitment times are synchronous across the nodes. For intermediate coupling, nodes 1, 2, 15 and 16 take a relatively longer time to recruit than the nodes in the center of the chain. These are edge artifacts of the coupling structure used and can be rectified by simulating with a longer chain (*N* ≫ 16) and taking the results from the central 16 nodes.
(EPS)

S18 Fig Modeling with linear gradient in excitability and constant fixed coupling *A* = 0.03; compare to Fig 5. Panel **a** shows the scaled recruitment times from data and model simulations with gradient in node excitability given by (*ν*^0^, *δν*) = (0.11, 0.004) indicated by the red stars in panels **b–c**. Panels **b** and **c** show the distance *d* and the proportion of ventral initiation *P*_*V*_, respectively. Here there is no overlap in the (*ν*^0^, *δν*) parameter space of the region that gives *d* ≤ 0.2 and within the confidence interval for *P*_*V*_.
(EPS)

S19 FigThe assignment of seizures to the fast, slow and multi-domino groups computed by submitting the first 8 seconds of each EEG recording epoch to the seizure detection algorithm.Panel **a** shows 26 seizures from one individual, 12 clinically classified as generalized onset (•) and 3 classified as having focal onset (⧫); with an additional 11 seizures from multiple subjects classified as having focal onset (⧫), with the lines $L_{1}^{\prime }$ and $L_{2}^{\prime }$; compare with Fig 1a. The three example seizures are shown in darker blue, purple and orange. The lines *L*′1 and *L*′2 are given by *L*′1 = 73.7624 + −24.1667*r* + −138.5209*l* and *L*′2 = 13.1003 + −9.35*r* + −42.6316*l*, where *r* is the recruitment time and *l* is the lag time. Panel **b** shows 1145 generalized seizures. The separation between groups in panel **a** is clearer than in Fig 1a, but the lag and recruitment times for the generalized seizure onset shown in panel **b** still form a spectrum across all three groups.(EPS)
