# Supplementary material for: Domino-like transient dynamics at seizure onset in epilepsy
Source: PLoS Comput Biol. 2020 Sep 28;16(9):e1008206. doi: 10.1371/journal.pcbi.1008206 (PMC7544071; doi:10.1371/journal.pcbi.1008206)
Supplement: S1 Text — (PDF) [file pcbi.1008206.s001.pdf]

Supplementary Material for “Domino-like transient dynamics at seizure onset in epilepsy”

Jennifer Creaser, Congping Lin, Thomas Ridler, Jonathan T Brown, Wendyl D’Souza, Udaya Seneviratne, Mark Cook, John R Terry, Krasimira Tsaneva-Atanasova.

Text 1

Association Index We compute the *association index* denoted $\mathcal{A}_{n,m} \in (0,1)$ that assigns a functional connection strength from node m to node n. For this we use the equation given in [1] as follows. For two time series x_i and y_i the association or h^2 index is given by

$$h^2(y|x) = 1 - \frac{\frac{a}{N}\sum_{a=1}^{M}\sum_{i,x_i\in B_a}(y_i - < y >_a)^2}{\text{std}^2(y)} \tag{1}$$

where

$$< y >_a = \frac{\sum_{i,x_i\in B_a} y_i}{N_a}, \ \sum_a Na = N.$$

Here, x_i are classified into M bins B_a for $a = 1, \ldots, M$ that contain N_a points. The summation in the numerator goes over all bins as well as all points in each bin. In our calculations, we use $M = 100$ bins.

References

1. Kalitzin SN, Parra J, Velis DN, and Lopes da Silva FH. Quantification of Unidirectional Nonlinear Associations Between Multidimensional Signals. IEEE Transactions on Biomedical Engineering. 2007; 54(3):454-61.
